# Assessment of oral hygiene habits, oral hygiene practices and tooth wear among fertilizer factory workers of Northern India: A Cross sectional study

**DOI:** 10.4317/jced.52332

**Published:** 2015-12-01

**Authors:** Vivek V. Gupta, Kailash Asawa, Nagesh Bhat, Mridula Tak, Salil Bapat, Pulkit Chaturvedi, Pradeep Philip-George, Neha Chitkara, Maulikkumar-Natubhai Patel, Kushal Shinde, Prabhjot-Kaur Sidhu

**Affiliations:** 1 MDS, Senior Lecturer, Dept. of public health dentistry, Maharaja Ganga Singh Dental College and Research Center, Sri Ganganagar, Rajasthan, India; 2 MDS, Reader, Dept. of public health dentistry, Pacific dental college and hospital, Debari, Udaipur, Rajasthan, India; 3 MDS, Professor and Head, Dept. of public health dentistry, Pacific dental college and hospital, Debari, Udaipur, Rajasthan, India; 4 MDS, Senior Lecturer, Dept. of Public health dentistry, Pacific dental college and hospital, Debari, Udaipur, Rajasthan, India; 5 MDS, Senior Lecturer, Dept. of Public health dentistry, SMBT Dental College and Hospital, Maharashtra, India; 6 MDS, Senior Lecturer, Dept. of Public health dentistry, R.R. Dental College and Hospital, Udaipur, Rajasthan, India; 7 MDS, Reader, Dept. of Orthodontics and Dentofacial Orthopedics, AL Azhar Dental College, Perumbillichira PO, Thodupuzha, Kerela; 8 MDS, Senior Lecturer, Dept. of Oral medicine and Radiology, Mahatma Gandhi dental college and hospital, Debari, Jaipur, Rajasthan, India; 9 MPH, University of North Texas Health Science Center; 10 MDS, Post graduate student, Dept. of Public Health Dentistry, Pacific Dental College and Hospital, Debari, Udaipur, Rajasthan, India; 11 BDS, Luxmi Bai Dental College and Hospital, Punjab, India

## Abstract

**Background:**

The association between oral hygiene habits & practices and severity of tooth wear lesion varies from community to community and also from occupation to occupation. The present study was conducted with to assess oral hygiene habits & practices and tooth wear among fertilizer factory workers of Punjab, India.

**Material and Methods:**

A descriptive cross sectional survey was conducted among 965 male workers who were aged between 19–58 years, who were the workers of fertilizers factory of Bathinda, India. An interview on the demographic profile, oral hygiene practices, and adverse habits followed a clinical examination for recording the Tooth Wear (Smith and Knight Index 1984) using Type III examination. The Chi–square test and a Stepwise multiple linear regression analysis were used for the statistical analysis. Confidence interval and p-value set at 95% and ≤ 0.05 respectively.

**Results:**

In the present study majority (47.2%) of the study population used chew sticks for cleaning their teeth. Overall prevalence of adverse habits was reported (92.4%). Study population showed higher prevalence of tooth wear (77.1%). Best predictors identified for Tooth Wear were oral hygiene practices, adverse habits, years of work experience and age respectively.

**Conclusions:**

Considerable percentages of fertilizer factory workers have demonstrated a higher prevalence of tooth surface loss. This may be useful in designing the investigations that aim to further explore the causes for these findings and more importantly to plan oral health promotion program implementing both preventive and curative strategies.

** Key words:**Tooth wear, smith & knight index, fertilizer factory.

## Introduction

Tooth wear is a natural consequence of ageing; the process can be considers pathological if the rate of loss is an excessive and cause aesthetic, functional or sensitivity problems (1). Tooth wear is a multi-factorial condition leading to the loss of enamel and dentine (2). Based upon the etiological factors, tooth wear has traditionally been divided into three categories: attrition, abrasion and erosion. The erosion is the most common and causes greatest damage (3).

Decline in caries rate in some countries, erosion is now becoming a focus of increasing interest both in clinical dentistry and research (4). Erosion is defined as the loss of tooth substance by a chemical process that does not involve bacterial action (5). The occurrence of erosion was reported as early as the 19th century and since then the incidence and prevalence of dental erosion is increasingly being reported (6).

Based upon the etiology dental erosion is termed extrinsic, intrinsic or idiopathic and according to the anamnesis the acids producing tooth destruction may be exogenous or unknown origin (7). Intrinsic and extrinsic acid loads determine the acidity levels of the oral cavity. If the pH goes beyond 5.5 (the threshold level for healthy enamel), (8,9) dental erosion may be triggered (7).

Initially, dental erosion appears as a smooth silky-shining glazed enamel surface. Further progression may lead to the development of shallow concavities or to rounding and grooving of the edges or the cusps of the tooth surfaces (10,11). In patients with severe dental erosion, the enamel is often totally removed, leaving a vulnerable dentin surface which is often associated with a painful sensitivity and is prone to further erosion and mechanical wear (12).

Dental erosion can be an occupational hazard (13). It is caused by exposure to various types of acidic contaminants in the work-place such as chemicals, petrochemicals, metals and semicounductor (14).

Occupational exposure to sulfuric acid and nitric acid mists has been described in association with dental erosion and ulcerative mucosal lesions (15) explained by the high irritant and corrosive acid effects that damage the enamel structure, cause inflammatory and immune reactions, and reduce the salivary pH that can also compromise resistance to infections in the oral cavity (16).

Fertilizer workers have been exposed to chemical vapors which lead to general and oral health problem. Despite the hazardous nature of their occupation very little research has been conducted and reported on fertilizer worker’s health and safety. In view of these observations, this study was conducted with the objective to assess oral hygiene habits, oral hygiene practices and tooth wear among fertilizer factory workers of Bathinda, Punjab, India.

The present study hypothesized that there is an association between fertilizer exposure and tooth wear among fertilizer factory workers.

## Material and Methods

The present study was conducted to assess the tooth wear among fertilizer factory workers of Bathinda, Punjab, India.

-Study design and study duration:

A cross sectional descriptive survey was conducted among workers of fertilizer factory in Bathinda city, Punjab, India. The duration of the study was from June 2013 – August 2013.

-Official Permission and Ethical clearance:

The study protocol was reviewed by the Ethical Committee of Pacific Dental College and Hospital ethical clearance and the official permission was obtained.

-Informed consent:

After explaining the purpose and details of the study, a written informed consent was obtained from all the subjects who were willing to participate.

-Inclusion criteria.

• Who were available at the time of the study 

• Voluntary participation.

-Exclusion criteria.

• Those not willing to participate in the study.

• Those who were working on daily wages in the factory.

• Those with any chronic illness or on medications.

-Questionnaire Design:

A survey proforma was designed with the help of WHO Oral Health Assessment (1997) (17) consisted of three sections:

1. General information: Demographic data including name, age, gender, date of birth, education and years of experience.

2. Questionnaire assessing information regarding oral health practices and adverse habits.

3. Clinical parameters: Clinical parameter assessed was Tooth wear.

-Training and Calibration

Before the commencement of the study, the examiner was standardized and calibrated in the Department of Public Health Dentistry by the Head of Department to ensure uniform interpretations, understanding, and application of the codes and criteria for the diseases to be observed and recorded and to ensure consistent examination. The examiner first practiced the examination on a group of 10 subjects with a wide range of levels of disease conditions. Then the examiner applied the diagnostic criteria by examining a group of 20 subjects, with full range of disease condition, twice on successive days. The intra examiner reliability for TWI (Tooth Wear Index) was assessed using Kappa statistics, which was found to be 90%.

-Pilot study 

A pilot study was carried out among 50 factory workers to determine the feasibility and practicability of the study and the time required for examination of each subject. It helped to know the practical difficulties while conducting the survey. Both questionnaire and indices interpretation was done on 50 factory workers. The prevalence of tooth wear was found to be 73%. The information obtained from participants of pilot study was excluded from the main study.

-Sampling design:

Before the commencement of the study, list of fertilizer factory workers was obtained from the office. As per the list, there were total 1056 workers. Among them, all the workers who gave informed consent were included in the study.

-Clinical assessment and data collection

The examiner visited the site on the predetermined dates according to the schedule. Authorities were requested to provide an area for examination with adequate illumination along with a table and chair. ADA type III examination was done by the investigator under natural daylight using mouth mirror and CPI probe with the study subjects seated on an ordinary chair.

In the proforma tooth surface loss of the study population was registered and scored according to Smith & Knight index 1984 (18).

The scoring used for the severity of tooth surface loss was as follows:

Score 0: No loss of enamel surface characteristics

Score 1: Loss of enamel surface characteristics

Score 2: Loss of enamel exposing dentine for less than one third of surface

Score 3: Loss of enamel exposing dentine for more than one third of surface

Score 4: Complete enamel loss–pulp exposure–secondary dentine exposure 

Score of the highest affected teeth in the upper and lower anterior sextants were considered as the score for the sextant.

Duplicate examinations were conducted on 5% (n=50) of the population during the course of the study with a kappa statistic of 95%. The subjects who needed emergency dental care were referred to the nearest dental facility available. Survey findings were reported to concerned authorities after the examination on the last day of visit to site. A total of 965 subjects gave a written informed consent and were examined.

-Statistical analysis

The recorded data was compiled and entered in a spreadsheet computer program (Microsoft Excel 2010) and then exported to data editor page of SPSS version 17 (SPSS Inc., Chicago, Illinois, USA).

Descriptive statistics included computation of percentages, means and standard deviations. The statistical tests applied for the analysis were Pearson’s chi-square test (χ2) and Stepwise multiple linear Regression analysis. For all tests, confidence interval and *p*-value were set at 95% and ≤ 0.05 respective.

## Results

 Table 1 depicts the distribution study population by demographic characteristics. Of the total 965 subjects who participated in the survey all were males. Majority of the study subjects were Hindus (n=449; 46.5%): Sikhs (n=378; 39.2%), Muslims (n=88; 9.1%) and others (50; 5.2%) formed the remaining population. Higher proportions of participants were married (71.9%) and had 5-10 years of work experience (45.2%). Only (12.7%) of the factory workers had education above middle level.

**Table 1:**
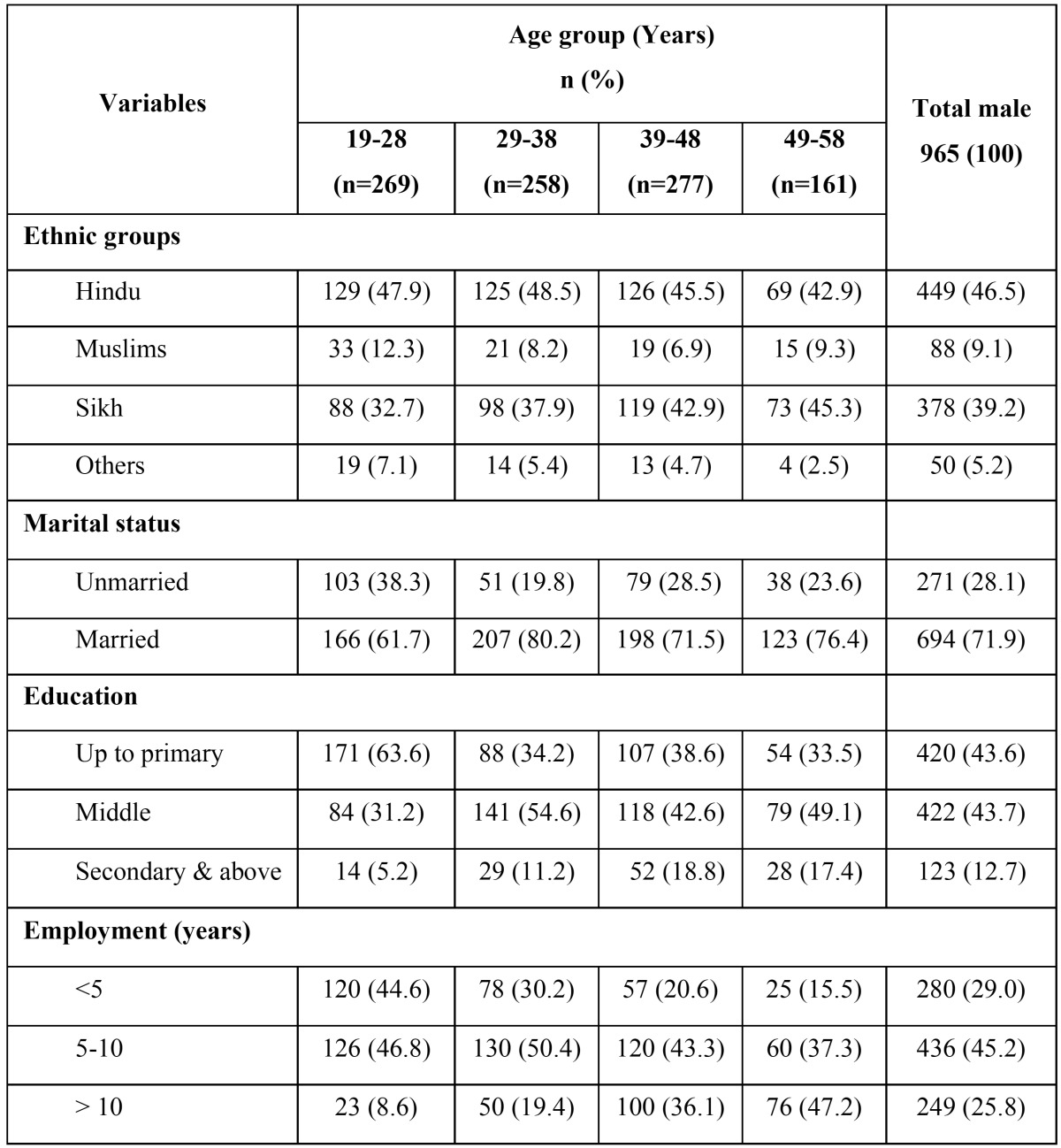
Distribution of study population by demographic characteristics.

 Table 2 shows that 39.4% of 19-28 years old subjects used tooth brush and tooth paste/tooth powder and 47.2% of 49-58 years old subjects used chewing sticks for cleaning their teeth. Majority (n=373; 38.6%) of the study population used chew sticks for cleaning their teeth. Use of toothbrush diminished significantly with increasing age from 19-28 years age group to 49-58 years age group (*p*=0.001). Overall prevalence of adverse habits among the study population was 92.4%. The prevalence of consumption of smoking tobacco, smokeless tobacco, combinations of smoking tobacco and smokeless tobacco, alcohol and combinations of tobacco and alcohol were 14.1%, 14.7%, 13.9%, 15.8% and 31.8% respectively. Adverse habits showed a significant rise with increasing age (*p*=0.001).

**Table 2:**
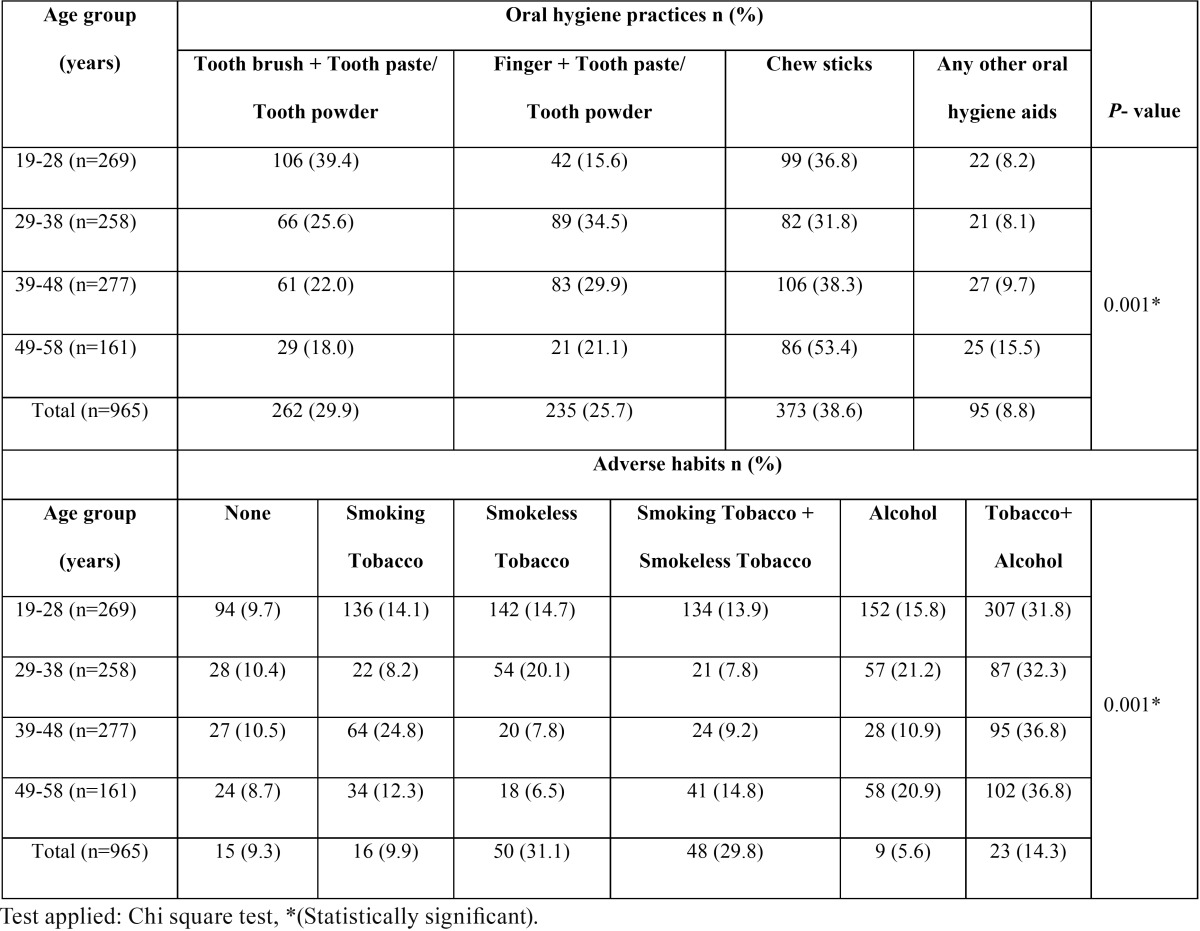
Distribution of oral hygiene practices and adverse habits among study population by age.

 Table 3: reveled tooth surface loss using Smith & Knight tooth wear index by age. Of the whole population, 745 (77.1%) of the subject had tooth surface loss. Score 2 and Score 3 tooth surface loss i.e. 32.6% and 36.7% was highly prevalent among the affected population. Score 3 tooth surface loss showed a significant increase with age (*p*=0.001). Overall prevalence for Score 0 was found to be 221 (22.9%), that of Score 1 were 62 (6.4), Score 2 were 315 (32.6), Score 3 were 354 (36.7) and for Score 4 it was 13 (1.4) respectively. With the increase in years of employment there was significant increase in score 1; score 2 and score 3 tooth surface loss was observed (*p*=0.001).

**Table 3:**
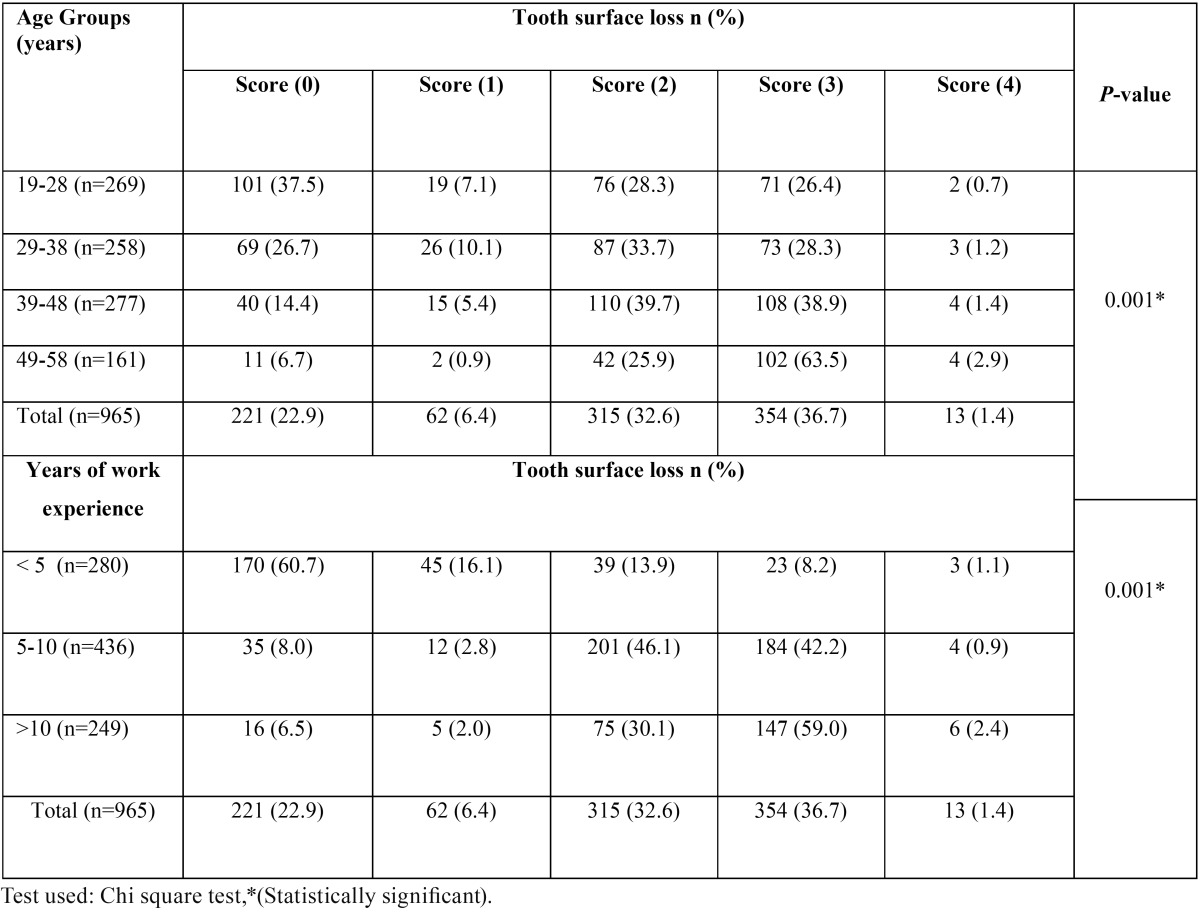
Distribution of the study population according to the scores of tooth surface loss using Smith & Knight tooth wear index by age and years of work experience.

 Table 4: Stepwise multiple linear regression analysis revealed the best predictors in the descending order for tooth wear were oral hygiene practices, adverse habits and years of employment and age with variances of 5.6%, 9.9%, 12.1% and 14.6% respectively.

**Table 4:**
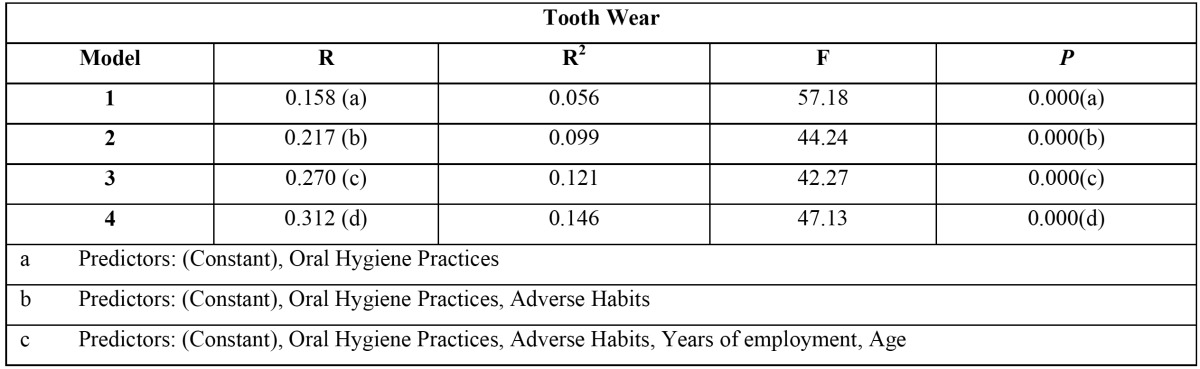
Stepwise Multiple Linear Regression analysis with Tooth Wear as a dependent variable.

## Discussion

This study of oral health status among fertilizer factory workers is a pioneer study by itself, so a direct comparison with studies conducted on other fertilizer factory worker population is difficult; thus an attempt is made to compare the study results with other populations similar in character.

The study population was in the age range of 19-58 years. Majority of the subjects were in the age range of 39-48 years with only a small proportion belonging to the age group of 49-58 years. Furthermore, demographics of the study population showed that major proportion of the study participants were Hindus (46.5%) approximately; three fourths were married and had 5-10 years of employment (45.2%). Only 12.7% of them had educational status above middle school level. This is in accordance with the findings of Petersen PE and Henmar P (2011) (19) who also reported a low level of education among Danish granite industry workers. It reveals that the fertilizer factory workers might not have adequate knowledge about oral and occupational health.

The most prevalent oral hygiene practices among the study population were chewsticks (38.6%) and finger with tooth paste/tooth powder (25.7%). Use of tooth brush was limited to 29.9% of the study population. This finding is in accordance with the findings of Green marble mine workers at Kesariyaji, Rajasthan, India (14) but is analogous to the results reported by Sakthi *et al.* (2011) at Chennai, India (19) where 36.9% of building construction workers used toothbrush and tooth paste for cleaning their teeth. Use of toothbrush was reported with high prevalence (39.4%) among 19-28 years age group than among older age groups up to 58 years which is in corroboration with the results of previous studies among various target groups (18,20). This finding may be attributed to the self-consciousness of adolescents who are more concerned about their image and grooming at this stage of development.

The present study elicited smokeless tobacco use of 14.7% which was much lesser than that reported by Ansari *et al.* (2010) (21) among power loom workers of Allahabad, India (66.1%). 15.8% of study population had a habit of alcohol and 31.8% used both tobacco and alcohol, which was little higher (26.3%), reported among green marble mine laborers of India (22) consuming alcohol. The present study demonstrated prevalence of tobacco usage was increasing subsequently in old age groups as compared to younger age groups. Townsend *et al.* (1994) (23) also portrayed a similar pattern and attributed this finding to the fact that young people generally have relatively low incomes with a high proportion of it available for discretionary expenditure, so that changes in income are more likely to affect their tobacco consuming patterns. It is clear from this cross-sectional study that tobacco usage and alcohol consumption is highly prevalent among fertilizer factory workers. The reasons underlying this may be low educational status, occupation involving hard labor, and poverty.

In the present study the presence of tooth surface loss of the anteriors was recorded and its severity was graded according to Smith & Knight index 1984 criteria. The prevalence of tooth surface loss was found to be 77.1%. This might be due to heavy and continuous acidic dust exposure and less use of personal protective measures (PPM) among factory workers. The results were in agreement with the previous study conducted at Tanza Cement Company Tanzania 14 (72.2%), Danish Granite industrial and workers exposed to olivine dust in Norway (100%) abrasion was found in the oral cavity in particular in the front teeth (8,24).

In the present study severity and prevalence of tooth surface loss increased with the duration of employment in the factory among factory employees. Similar finding was reported in a previous study conducted at Tanza cement company Tanzania and in Danish Granite industries in which there was an increased severity of tooth surface loss with length of service of the workers in the factory (13,7).

An analytical study design is needed to observe the association between tooth surface loss and acidic dust exposure in fertilizer factory, because tooth surface loss can be attributable to other causes also.

## Conclusion

The findings of the study provides with some insight into the oral diseases (tooth wear) of fertilizer factory worker’s population. Considerable percentages of fertilizer factory workers have demonstrated a higher prevalence of tooth surface loss. This may be useful in designing the investigations that aim to further explore the causes for these findings and more importantly to plan oral health promotion program implementing both preventive and curative strategies.
